# Single-Use Disposable Electrochemical Label-Free Immunosensor for Detection of Glycated Hemoglobin (HbA1c) Using Differential Pulse Voltammetry (DPV)

**DOI:** 10.3390/s16071024

**Published:** 2016-07-01

**Authors:** Alireza Molazemhosseini, Luca Magagnin, Pasquale Vena, Chung-Chiun Liu

**Affiliations:** 1 Dipartimento Chimica Materiali e Ingegneria Chimica “Giulio Natta”, Politecnico di Milano, Via Mancinelli 7, 20131 Milan, Italy; axm1058@case.edu (A.M.); luca.magagnin@polimi.it (L.M.); pasquale.vena@polimi.it (P.V.); 2 Department of Chemical & Biomolecular Engineering and Electronics Design Center, Case Western Reserve University, 10900 Euclid Avenue, Cleveland, OH 44106, USA

**Keywords:** immunosensor, HbA1c, differential pulse voltammetry, 3-MPA

## Abstract

A single-use disposable in vitro electrochemical immunosensor for the detection of HbA1c in undiluted human serum using differential pulse voltammetry (DPV) was developed. A three-electrode configuration electrochemical biosensor consisted of 10-nm-thin gold film working and counter electrodes and a thick-film printed Ag/AgCl reference electrode was fabricated on a polyethylene terephthalate (PET) substrate. Micro-fabrication techniques including sputtering vapor deposition and thick-film printing were used to fabricate the biosensor. This was a roll-to-roll cost-effective manufacturing process making the single-use disposable in vitro HbA1c biosensor a reality. Self-assembled monolayers of 3-Mercaptopropionic acid (MPA) were employed to covalently immobilize anti-HbA1c on the surface of gold electrodes. Electrochemical impedance spectroscopy (EIS) and X-ray photoelectron spectroscopy (XPS) confirmed the excellent coverage of MPA-SAM and the upward orientation of carboxylic groups. The hindering effect of HbA1c on the ferricyanide/ferrocyanide electron transfer reaction was exploited as the HbA1c detection mechanism. The biosensor showed a linear range of 7.5–20 µg/mL of HbA1c in 0.1 M PBS. Using undiluted human serum as the test medium, the biosensor presented an excellent linear behavior (R^2^ = 0.999) in the range of 0.1–0.25 mg/mL of HbA1c. The potential application of this biosensor for in vitro measurement of HbA1c for diabetic management was demonstrated.

## 1. Introduction

HbA1c is a stable glycosylated hemoglobin formed by the non-enzymatic reaction of glucose with the N-terminal valine of the β-chain of normal adult hemoglobin (HbA0) [1,2,3]. The HbA1c level is defined as the ratio between the HbA1c concentration and the total hemoglobin concentration. It is considered as a diagnostic biomarker for diabetic patients in addition to the measurement of the blood glucose level. The clinical reference range of HbA1c to the total hemoglobin (HbA0) is 5%–20%, and a value of 4%–6.5% is considered normal [4]. The blood glucose level of a diabetic is not very stable, even over one single day, and thus the measurement of the HbA1c level can provide a more accurate indication of the glucose level in the blood over a time period of eight to 12 weeks. Therefore, the measurement of the HbA1c level is important for the long-term control of the glycemic state in diabetic patients [5].

HbA1C as the biomarker of diabetes can capture chronic hyperglycemia better than the oral glucose tolerance test (OGTT) or fasting plasma glucose (FPG) evaluation. Therefore, HbA1c can be a robust biomarker for both diagnosing and monitoring diabetes. On the other hand, arguments for defining diabetes by high blood glucose rather than by glycation of proteins persist. Also, the detection of HbA1c remains relatively costly. Thus, a cost-effective single-use disposable HbA1c biosensor that can measure the HbA1c level will be highly desirable for diabetic patient management [1].

There are clinical methods to analyze HbA1c including ion-exchange and boronated affinity chromatography [6,7], electrophoresis [8] and fluorescence [9,10]. However, these methods are relatively expensive, and require pretreatment of the blood sample, extensive analysis time and a skillful operator. Thus, a single-use disposable in vitro biosensor capable of the measurement of HbA1c with a suitable sensitivity and selectivity for diabetic management is scientifically and clinically significant. In addition to having the sensitivity and specificity of detecting HbA1c in a meaningful physiological range, it should require a small sample volume such as 10–15 µL of blood or other physiological fluids and it needs to have a fast response time, such as in seconds. Thus, this HbA1c biosensor can serve as a stand-alone monitoring system for HbA1c or as a part of a double diagnostic system for measuring the HbA1c and blood glucose simultaneously.

Electrochemical HbA1c biosensors have been constructed by modifying the surface of an electrode element by sugar-binding materials (mostly boronic acid), proteins and antibodies specific to HbA1c. This bio-recognition mechanism is followed by a transduction mechanism including amperometry/voltammetry, potentiometry or impedometry [11]. Zhou et al. [12] described an electrochemical interface of BPA-PQQ/ERGO on glassy carbon electrodes for the detection of HbA1c using differential pulse voltammetry (DPV). However, the fabrication involved multiple steps and the electrode was not disposable. Furthermore, the selectivity of the biosensor toward HbA1c was only fair as a result of using a boronic acid derivative which has an affinity for all types of sugars and glycated proteins and is not limited only to HbA1c. Chopra et al. [13] employed a sandwich electrochemical immunoassay format which was developed on a screen-printed gold electrode using MPBA-SAM as the capture molecule and ferrocene labeled anti-HbA1c as a tracer. Kim et al. [14] exploited the catalytic property of HbA1c for H_2_O_2_ reduction to measure the HbA1c level. The sensor construction involved multiple steps of electrodeposition and potential cycling. Furthermore, it used a boronic acid–derivative capturing probe (aminophenyl boronic acid), and it required an additional step of blood sample pretreatment to remove glucose and other glycated proteins. Electrochemical impedance spectroscopy (EIS) for HbA1c detection was also suggested [15,16,17]. However, EIS requires sophisticated instruments and a long measuring time compared to amperometry/voltammetry techniques. EIS also involves an additional step of equivalent circuit modeling after data acquisition.

**Figure 1:**
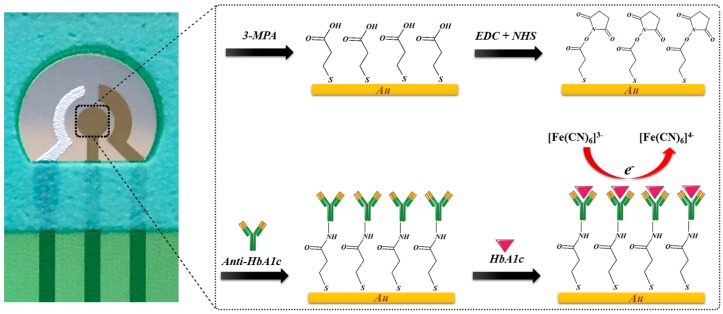
Schematic representation of the stepwise fabrication process of the immunosensor.

In this study, single-use disposable thin-film gold-based working and counter electrodes were constructed as a label-free HbA1c biosensor. Figure 1 shows a schematic representation of the fabrication steps of this biosensor. Anti-HbA1c was used as a selective HbA1c-capturing probe. Self-assembled monolayers of MPA were employed to covalently immobilize anti-HbA1c on the surface of the gold electrode. Differential pulse voltammetry (DPV) was employed as the electrochemical detection method to enhance the sensitivity through minimization of the charging current. Details of the fabrication processing of the biosensor will be given later.

## 2. Materials and Methods

### 2.1. Apparatus and Reagents

Phosphate Buffer Solution (PBS) 1.0 M (pH 7.4), human serum, 3-Mercaptopropionic acid (MPA), human hemoglobin, bovine serum albumin (BSA), *N*-(3-dimethylaminopropyl)-*N*′-ethylcarbodiimide hydrochloride (EDC) and *N*-hydroxysuccinimide (NHS) were purchased from Sigma-Aldrich (St. Louis, MO, USA). Human Hemoglobin A1c (HbA1c) and mouse anti-Human Hemoglobin A1c (anti-HbA1c) IgG1 were purchased from US Biological (Salem, MI, USA). Potassium hydroxide pellets, concentrated H_2_SO_4_ 95.0 to 98.0 w/w % and concentrated HNO_3_ 70% w/w % were received from Fisher Scientific (Pittsburgh, PA, USA). All the chemicals were used without further purification. A CHI660C (CH Instrument, Inc., Austin, TX, USA) Electrochemical Workstation was used for DPV and EIS investigations. All the experiments were conducted at room temperature. X-ray Photoelectron Spectroscopy (XPS) was performed by a PHI Versaprobe 5000 Scanning X-ray Photoelectron Spectrometer.

### 2.2. Electrode Fabrication

**Figure 2:**
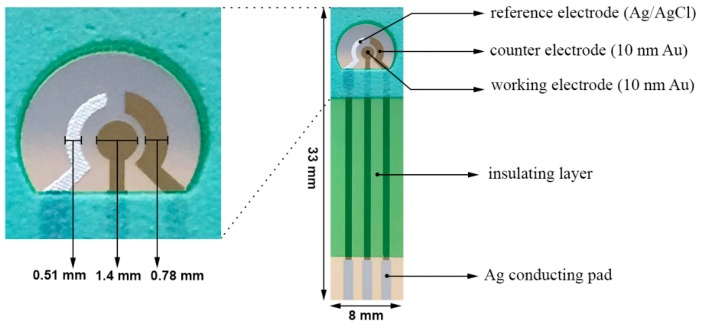
Structure and dimensions of the thin-film gold-based HbA1C biosensor prototype.

Figure 2 shows the biosensor prototype and its actual dimensions used in this study. The conventional three-electrode configuration consisted of a 10-nm-thin gold film used as working and counter electrodes and a thick-film printed Ag/AgCl reference electrode. Thin gold film was deposited on polyethylene terephthalate (PET) by sputtering technique without any binder and the biosensor was patterned by laser ablation technique. Separate masks were used producing different elements of the biosensor prototype. The Ag/AgCl reference electrode and the insulation layer were thick-film printed using DuPont #5870 Ag/AgCl and Nazdar APL 34 silicone-free dielectric inks respectively. 100 individual biosensors in 4 rows were fabricated on each PET sheet (355 × 280 mm^2^). The overall dimensions of an individual biosensor were 33.0 × 8.0 mm^2^. The working electrode area was 1.54 mm^2^ accommodating 10–15 µL of liquid test sample. The combination of sputtering and laser ablation techniques resulted in producing a very thin and yet uniform gold layer featuring high-reproduction and low-cost at the same time. This promising and unique fabrication technique allowed for mass production of single-use disposable biosensors. More detailed explanation of the electrode fabrication process can be found elsewhere [18].

### 2.3. Electrode Functionalization

#### 2.3.1. Pretreatment of Gold Electrode (AuE)

A pretreatment procedure based on those described previously [19,20] was applied to the gold electrode, prior to the MPA-SAM deposition. This three-step pretreatment procedure resulted in a significant decrease in electrode charge transfer resistance enhancing the reproducibility of the biosensor. A row of five or seven biosensors were immersed in a 2 M KOH solution for 15 min. After rinsing with copious amount of DI water, the biosensors were placed in a 20-fold diluted concentrated H_2_SO_4_ solution (95.0 to 98.0 w/w %) for another 15 min. DI water was then used to rinse the biosensor prototypes. The biosensors were then placed in a 20-fold diluted concentrated HNO_3_ solution (70% w/w %) for another 15 min. The biosensors were rinsed once more time with DI water and dried in a steam of nitrogen. During this pretreatment procedure, the counter and the reference electrodes were not covered. Concentrations of acids and base solutions used in this pretreatment procedure were optimized to be effective while maintained the integrity of the thin gold film working and counter electrodes and the Ag/AgCl reference electrode as well as the overall structure of the biosensor. The effectiveness of the pretreatment procedure was assessed using EIS and the results were excellent.

#### 2.3.2. Anti-HbA1c Immobilization on AuE

In all the electrode surface modification steps, both the counter and the reference electrodes were not covered resulting to a more practical and fast surface modification protocol. Typically, a row of five biosensors were subjected at once to surface modification. Self-assembled monolayers of MPA were exploited to wire the anti-HbA1c to the surface of gold working electrode. MPA molecule consisted of a thiol functional group at one end which processed a great affinity to gold and a carboxylic group at another end which was suitable for bonding covalently to proteins through peptide bond after an activation procedure. Thiol modification of gold electrode surface for protein immobilization was a well-developed technique [13,15,21]. The biosensor used in this study was immersed in 1 mM solution of MPA in ethanol for 24 h in dark, rinsed with DI water and dried in a steam of N_2_. The MPA modified AuEs were incubated in 0.1 M PBS (pH = 7.4) containing 0.25 M EDC and 0.05 M NHS for 5 h to activate MPA carboxylic groups. Activated AuEs were then rinsed by 0.1 M PBS and dried by N_2_ flow. 5 µL of 0.05 mg/mL anti-HbA1c was casted on the sensing area of each AuE and left to dry overnight at 4 °C. Antibody immobilized biosensors were rinsed with 0.1 M PBS and immersed in 1% BSA solution in 0.1 M PBS for 1 h to prevent non-specific bonding. The biosensors were then rinsed with 0.1 M PBS, dried under a steam of N_2_ and stored at 4 °C.

### 2.4. Electrochemical Measurements

**Figure 3:**
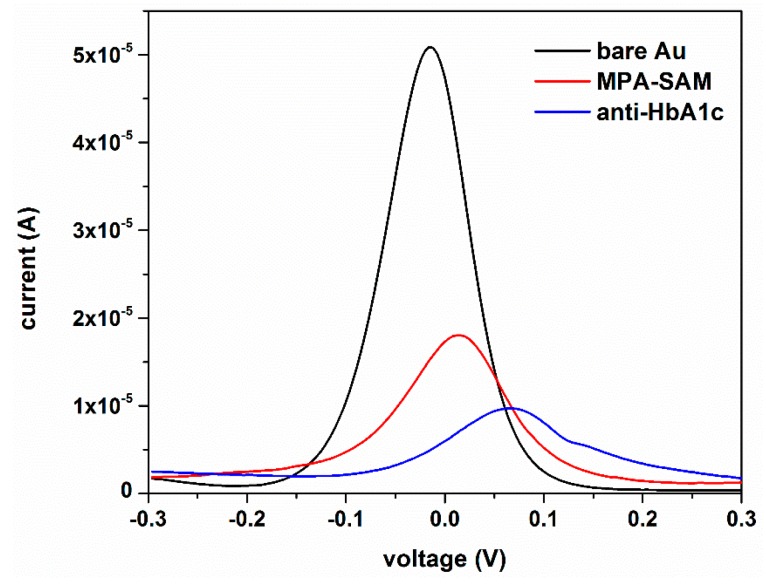
Gradual decrease in the signal generated by K_3_Fe(CN)_6_/K_4_Fe(CN)_6_ redox couple reaction as a result of MPA-SAM formation and anti-HbA1c (50 µg/mL in 0.1 M PBS) immobilization on the surface of gold electrode.

The experimental measurements were performed at ambient temperature. A solution of K_3_Fe(CN)_6_ and K_4_Fe(CN)_6_, with 5 mM in each component, was prepared in 0.1 M PBS and used as the redox coupled probe for DPV and EIS tests. EIS tests were performed in frequency range of 10^−2^ to 10^4^ Hz with 5 mV voltage amplitude. Randles equivalent circuit models were used to fit the Nyquist plots of EIS using EC-lab standard software. Anti-HbA1c immobilized AuEs were rinsed with 0.1 M PBS and dried in a stream of N_2_. 5 µL of HbA1c of selected concentration was pipetted on the sensing area of AuE and allowed to be dried for 2 h at room temperature. The biosensor was then rinsed with 0.1 M PBS. DPV and EIS measurements were performed after drop casting of 20 µL K_3_Fe(CN)_6_/K_4_Fe(CN)_6_ redox couple solution on the sensing area of AuE. EIS was used to investigate the surface coverage of MPA-SAM formed on AuE. The electron transfer reaction associated with ferricyanide/ferrocyanide redox couple transformation can be hindered by the presence of bulky moieties on the surface of the electrode. Figure 3 presents the gradual decrease in the signal generated by K_3_Fe(CN)_6_/K_4_Fe(CN)_6_ redox couple reaction as a result of MPA-SAM formation and Anti-HbA1c immobilization on the surface of gold electrode. Therefore, the hindering effect of HbA1c on ferricyanide/ferrocyanide electron transfer reaction was exploited as HbA1c detection mechanism.

### 2.5. X-ray Photoelectron Spectroscopy

The interaction between the MPA-SAM thiol groups and thin gold film electrode was investigated using X-ray photoelectron spectroscopy (XPS). The pretreated biosensor was immersed in 1 mM solution of MPA in ethanol for 24 h in dark, and then rinsed with DI water and dried in a stream of nitrogen. High resolution C(1s) and S(2p) spectra were collected using a monochromatic Al Kα X-ray source at the take-off angles of 10°, 50° and 90°. High resolution C(1s) spectra was used to assess the orientation of MPA-SAM molecules formed on AuE. The atomic ratio between MPA carboxylic group carbon (O–C=O) and the carbons from MPA hydrocarbon backbone (C–C) was calculated at each take-off angle and compared to verify the upward orientation of MPA-SAM.

## 3. Results and Discussion

### 3.1. MPA-SAM Characterization

#### 3.1.1. X-ray Photoelectron Spectroscopy

**Figure 4:**
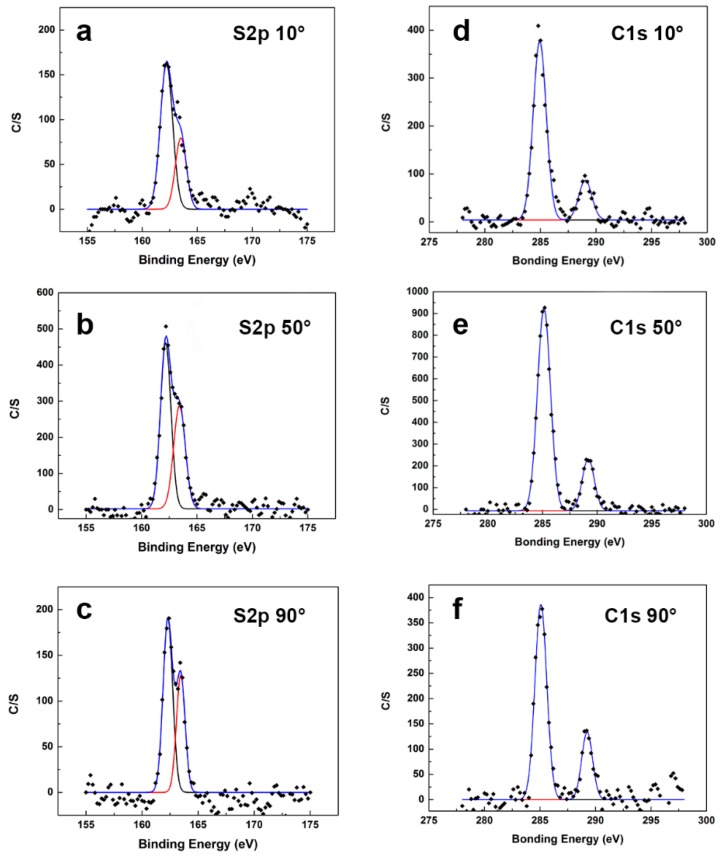
High resolution spectra of C(1s) and S(2p) obtained for MPA-SAM–modified AuE at take-off angles of 10°, 50° and 90°.

Figure 4 shows XPS high resolution spectra of C(1s) and S(2p) obtained for MPA-SAM–modified AuE at the take-off angles of 10°, 50° and 90°. The higher energy peak in S(2p) spectra was at 163.5 eV as presented in Figure 4a–c, representing the characteristic of the free thiol group (–SH) [22]. As a result of the Au–S covalent bond, the S(2p) peak was shifted by 1.5 eV negative of the 163.5 eV. Our results agreed exceptionally well with other reported research [22,23,24,25]. Thus, the higher intensity peak at 162 eV in Figure 4a–c confirmed the formation of the covalent bond between the MPA-SAM thiol groups and AuE. Furthermore, the relative intensity of the free thiol groups to covalently bonded thiol groups increased by increasing the take-off angle from 10° to 90°, as shown in Figure 4a–c. This indicated that by approaching toward the surface of the gold electrode, the number of free thiol groups appeared to decrease. The lower energy peak at the C(1s) spectra (285 eV) in Figure 4d–f was characteristic of saturated hydrocarbons (C–C) which could be assigned to carbons participating in the MPA backbone. The lower intensity peak at 288.9 eV in the C(1s) spectra was associated with –COOH [22]. Table 1 presents the atomic ratio between the MPA carboxylic group carbon (O–C=O) and the carbons from the MPA hydrocarbon backbone (C–C) at different take-off angles. This was calculated by the peak integration of the C(1s) spectra. As shown in Table 1, the relative number of carbons participating in the carboxylic groups decreased with decreasing the take-off angle from 90° to 10°. Thus, there were fewer numbers of carboxylic groups near the surface. This observation confirmed the upward orientation of MPA-SAM carboxylic groups in this MPA-SAM arrangement.

#### 3.1.2. EIS Assessment of MPA-SAM Surface Coverage

**Figure 5:**
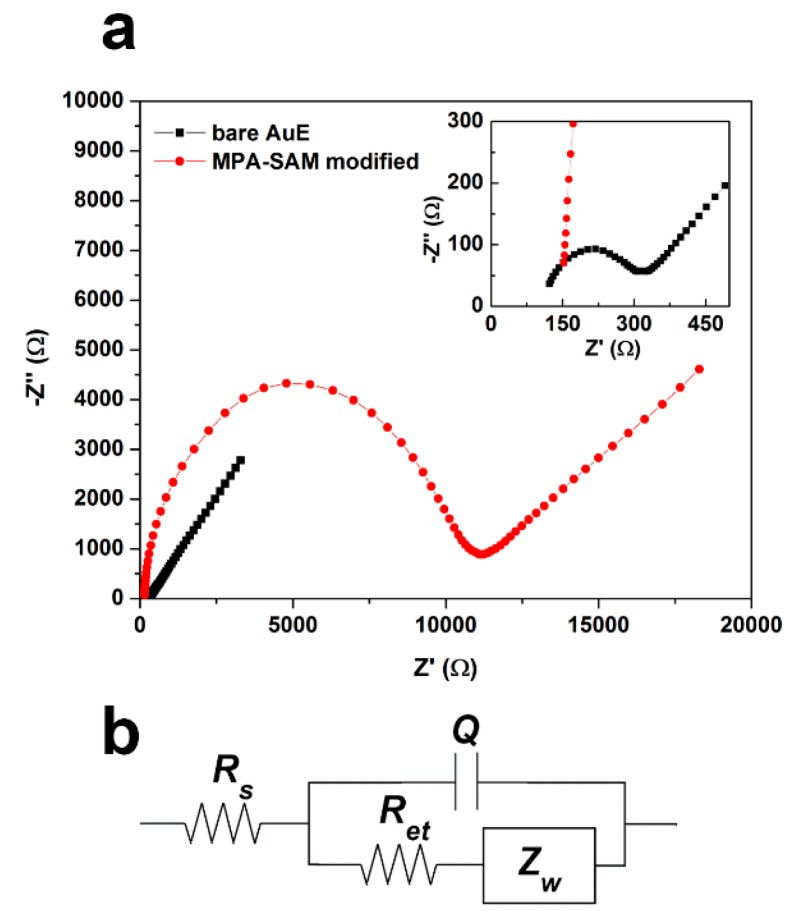
(**a**) Nyquist plots obtained for the bare and MPA-SAM-covered AuEs in a frequency range of 10^−2^ to 10^4^ Hz; (**b**) Randles equivalent circuit used to model the experimental data.

Figure 5a presents the electrochemical impedance spectroscopy (EIS) results for the bare and MPA-SAM–modified gold electrodes in the frequency range of 10^−2^ to 10^4^ Hz with a 5 mV voltage amplitude in the form of a Nyquist plot. Figure 5b shows the Randles equivalent circuit used to model the experimental data. Each component in the equivalent circuit represented an element in the physical electrode/electrolyte interface. The semicircular region of the Nyquist plot was associated with the electron transfer process which was modeled by a parallel circuit representation of a resistor ($R_{ct}$) and a capacitor (Q). The tail at the lower frequencies indicated the presence of a diffusion-limited electrochemical process, which was represented using the Warburg element ($Z_{w}$). The solution resistance was represented by $R_{s}$. The electrode charge transfer resistance ($R_{ct}$) was related to the MPA-SAM surface coverage assuming that the electron transfer reaction occurred only at uncovered spots and that the diffusion to these defects is planar [26,27]. The MPA-SAM surface coverage ratio was then calculated using the following equations:

$$\theta_{IS}^{R}=1-\left(\frac{R_{ct}^{AuE}}{R_{ct}^{SAM}}\right)$$

where $R_{ct}^{AuE}$ and $R_{ct}^{SAM}$ are charge transfer resistances measured at bare and MPA-SAM–covered electrodes, respectively. When $\theta_{IS}^{R}$ > 0.9, the surface coverage fraction could be evaluated using a model based on pinhole size:

$$\theta_{IS}^{P}=1-\left(\frac{\sigma_{w}}{m-\sigma_{w}}\right)$$

where $\sigma_{w}$ is the Warburg coefficient (slop of $Z^{\prime }$ vs. $\omega^{-1/2}$ plot obtained for bare AuE) and m is the slope of the linear interval in the high frequency region of the $Z^{\prime }$ vs. $\omega^{-1/2}$ plot obtained at MPA-SAM–modified AuE. Table 2 presents data obtained from the Randles equivalent circuit modeling of EIS Nyquist plots for bare and monolayer-covered electrodes. The calculated value of $\theta_{IS}^{P}$ = 0.9950 for MPA monolayers indicated a high coverage fraction compared to previous reports [26,27].

The high surface coverage of MPA-SAM together with the formation of the Au-S covalent bond and the upward orientation of the MPA carboxylic groups suggested that our immobilization process of anti-HbA1c on the MPA-SAM–modified gold electrode was very effective.

### 3.2. HbA1c Detection Using Differential Pulse Voltammetry (DPV)

The MPA-SAM–modified gold electrode was successfully characterized, and the HbA1c biosensor was ready for the evaluation of its performance. Cyclic voltammetry and amperometry are the two most used electrochemical detection techniques. However, differential pulse voltammetry (DPV) provides a linear sweep voltammetry with a series of regular voltage pulses superimposed on the linear potential sweep. Consequently, the current is measured immediately before each potential change. Thus, the effect of the charging current is minimized, achieving a higher sensitivity. Hence, in this study, we used DPV measurement to achieve higher sensitivity compared to cyclic voltammetry and amperometry.

#### 3.2.1. HbA1c Detection in 0.1 M PBS

**Figure 6:**
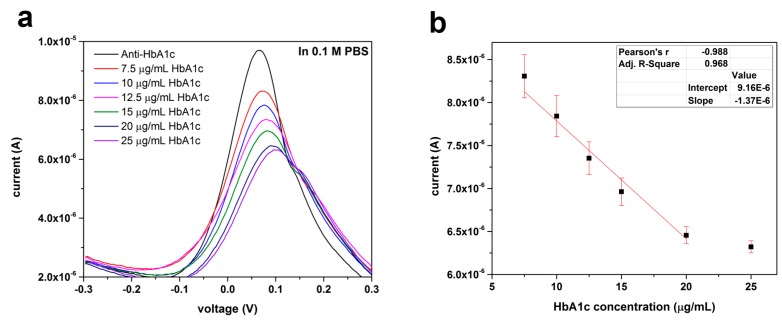
(**a**) DPV measurement of HbA1c antigen in 0.1 M PBS in the concentration range of 7.5–25 µg/mL using 5 µL of 0.05 mg/mL anti-HbA1c as a detection probe; (**b**) Calibration curve of HbA1c antigen concentration using the peak current output of the biosensor obtained from results of Figure 6a (*n* = 4).

Figure 6a shows the testing results of our HbA1c biosensor in 0.1 M PBS test medium over an HbA1c concentration range of 7.5–25 µg/mL. The DPV of the anti-HbA1c casted biosensor without HbA1c antigen was measured as the baseline. Each biosensor was used once for testing each HbA1c concentration, aimed at single-use disposable in vitro applications. Multiple test runs were carried out with *n* > 3. As can be seen in Figure 6a, there is a gradual decrease in the signal generated by the ferricyanide/ferrocyanide transformation reaction as a result of the increasing HbA1c concentration. This is due to the electron transfer hindering effect of the antigen which was captured on the surface of the anti-HbA1c immobilized gold electrode. Figure 6b is the calibration curve of HbA1c measurement in 0.1 M PBS test medium based on the testing results from Figure 6a. An acceptable correlation coefficient of 0.968 was obtained for the biosensor in the range of 7.5–20 µg/mL. As such, 7.5 µg/mL was the lowest detection limit for the biosensor in 0.1 M PBS testing medium as the sensor’s responses to antigen concentrations less than 7.5 µg/mL were not reproducible.

#### 3.2.2. HbA1c Detection in Undiluted Human Serum

**Figure 7:**
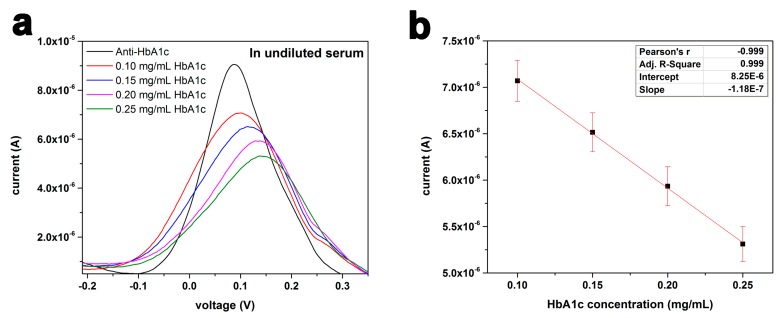
(**a**) DPV measurement of HbA1c antigen in serum in the concentration range of 0.10–0.25 mg/mL using 5 µL of 0.5 mg/mL anti-HbA1c as the detection probe of the biosensor; (**b**) Calibration curve of HbA1c antigen concentration in serum using the peak current output of the biosensor obtained from results of Figure 7a (*n* = 4).

HbA1c measurements were performed in undiluted human serum to show the potential application of the sensor in real blood samples. Considering the normal adult human hemoglobin (HbA1_0_) concentration of 150 mg/mL [28], the HbA1c concentration for a diabetic patient (6.5% HbA1c) is more than 9 mg/mL in whole blood. Samples with an HbA1c concentration range of 0.1–0.25 mg/mL were prepared in undiluted human serum which were 10 times higher than the ones that were tested in 0.1 M PBS, and these was more clinically relevant. The antibody concentration for the biosensors tested in serum was 0.5 mg/mL in 0.1 M PBS. Figure 7a shows the DPV measurements of the HbA1c antigen in undiluted serum over the concentration range of 0.10–0.25 mg/mL and it also includes the measurement of 0 mg/mL HbA1c serving as the baseline.

The biosensors for the measurement of HbA1c in serum were used only once for each HbA1c concentration to accomplish the goal of developing a single-use disposable in vitro HbA1c biosensor. Figure 6a,b exhibit the excellent performance of the biosensor for HbA1c detection in serum. The correlation coefficient for linear fitting was 0.999. Measurements of the HbA1c antigen in serum at the concentration level of µg/mL were also undertaken (data not presented). The DPV measurement of the HbA1c antigen in serum at the level of 15–25 µg/mL was good and meaningful. However, the repeatability of the DPV measurements at lower HbA1c antigen concentrations <10 µg/mL were only fair. This might be the lowest detection limit of HbA1c in serum. Nevertheless, the DPV measurements of HbA1c in undiluted serum over the range of 0.10–0.25 mg/mL demonstrated this potential application of the biosensor in diabetic management.

## 4. Conclusions

A single-use disposable in vitro HbA1c biosensor was designed, fabricated and produced in a cost-effective manner. The interaction between anti-HbA1c and its antigen (the analyte) was the bio-recognition mechanism of this biosensor. Differential pulse voltammetry (DPV) was employed as the transduction mechanism for this biosensor. Covalent immobilization of anti-HbA1c onto the gold thin-film electrode was accomplished by MPA-SAM modification. Confirmation of the thiol group bonding on the gold-based electrode elements and the upward orientation of the MPA-SAM carboxylic groups were experimentally assessed. Excellent coverage and upward orientation of the MPA-SAM was obtained. DPV measurements of HbA1c in 0.1 M PBS test medium in the range of 7.5–20 µg/mL and in serum in the range of 0.1–0.25 mg/mL were carried out. The results were excellent. This research suggested that a cost-effective, single-use, disposable in vitro HbA1c biosensor could be used alone or together with a blood glucose biosensor for better diabetic management applications.

## Figures and Tables

**Table 1:** Atomic ratio between MPA carboxylic group carbon (O–C=O) and the carbons from the MPA hydrocarbon backbone (C–C) at different take-off angles.

Take-off Angle	–COOH Count	O–C=O Count	–COOH/O–C=O Atomic Ratio
10°	122.20	547.62	0.2231
50°	330.02	1385.55	0.2382
90°	164.08	516.98	0.3174

**Table 2:** Data obtained from Randles equivalent circuit modeling of EIS Nyquist plots for bare and monolayer-covered electrodes.

Surface	Q(µF)	$Z_{w}\left(\Omega \right)$	$\sigma_{w}\left(\Omega \cdot s^{-1/2}\right)$	m	$R_{ct}\left(\Omega \right)$	$R_{s}\left(\Omega \right)$	$\theta_{IS}^{R}$	$\theta_{IS}^{P}$
bare AuE	1.31	972.6	367.7		201.5	107		
MPA-SAM	0.46	1738		74,542	10,347	152	0.9805	0.9950

## References

[1] Goldstein D.E., Little R.R., Lorenz R.A., Malone J.I., Nathan D., Peterson C.M., Sacks D.B. (2004). Tests of glycemia in diabetes. Diabetes Care.

[2] Kilpatrick E.S. (2004). HbA1c measurement. J. Clin. Pathol..

[3] John W.G. (2006). Haemoglobin A1c reference method. Scand. J. Clin. Lab. Investig..

[4] John W.G. (2003). Haemoglobin A1c: Analysis and standardisation. Clin. Chem. Lab. Med..

[5] Jeppsson J.O. (2002). Approved IFCC Reference Method for the Measurement of HbA1c in Human Blood. Clin. Chem. Lab. Med..

[6] Eckerbom S., Bergqvist Y., Jeppsson J.O. (1994). Improved method for analysis of glycated haemoglobin by ion exchange chromatography. Ann. Clin. Biochem..

[7] Frantzen F., Grimsrud K., Heggli D.E., Faaren A.L., Løvli T., Sundrehagen E. (1997). Glycohemoglobin filter assay for doctors’ offices based on boronic acid affinity principle. Clin. Chem..

[8] Zhao Z., Basilio J., Hanson S., Little R.R., Sumner A.E., Sacks D.B. (2015). Evaluation of hemoglobin A1c measurement by Capillarys 2 electrophoresis for detection of abnormal glucose tolerance in African immigrants to the United States. Clin. Chim. Acta.

[9] Yang W., Yan J., Springsteen G., Deeter S., Wang B. (2003). A novel type of fluorescent boronic acid that shows large fluorescence intensity changes upon binding with a carbohydrate in aqueous solution at physiological pH. Bioorg. Med. Chem. Lett..

[10] Kataoka K., Hisamitsu I., Sayama N., Okano T., Sakurai Y. (1995). Novel Sensing System for Glucose Based on the Complex Formation between Phenylborate and Fluorescent Diol Compounds. J. Biochem..

[11] Wang B., Anzai J. (2015). Recent Progress in Electrochemical HbA1c Sensors: A Review. Materials.

[12] Zhou Y., Dong H., Liu L., Hao Y., Chang Z., Xu M. (2014). Fabrication of electrochemical interface based on boronic acid-modified pyrroloquinoline quinine/reduced graphene oxide composites for voltammetric determination of glycated hemoglobin. Biosens. Bioelectron..

[13] Chopra A., Rawat S., Bhalla V., Suri C.R. (2014). Point-of-Care Amperometric Testing of Diabetic Marker (HbA1c) Using Specific Electroactive Antibodies. Electroanalysis.

[14] Kim D.M., Shim Y.B. (2013). Disposable amperometric glycated hemoglobin sensor for the finger prick blood test. Anal. Chem..

[15] Park J.-Y., Chang B.-Y., Nam H., Park S.-M. (2008). Selective Electrochemical Sensing of Glycated Hemoglobin (HbA(1c)) on Thiophene-3-Boronic Acid Self-Assembled Monolayer Covered Gold Electrodes. Anal. Chem..

[16] Halámek J., Wollenberger U., Stöcklein W., Scheller F.W. (2007). Development of a biosensor for glycated hemoglobin. Electrochim. Acta.

[17] Bhat N., Srinivasan S. Detection of GlycatedHemoglobin using 3-AminoPhenylboronic acid modified Graphene Oxide. Proceedings of the IEEE/NIH Life Science Systems and Applications Workshop (LiSSA).

[18] Janyasupab M., Lee Y., Zhang Y., Liu C.W., Cai J., Popa A., Samia A.C., Wang K.W., Xu J., Hu C.-C. (2015). Detection of Lysyl Oxidase-Like 2 (LOXL2), a Biomarker of Metastasis from Breast Cancers Using Human Blood Samples. Curr. Biomark..

[19] Willner I., Riklin A., Wlllner I., Rlklln A. (1994). Electrical Communication between Electrodes and NAD(P)+-Dependent Enzymes Using Pyrroloquinolinequinone-Enzyme Electrodes in a Self-Assembled Monolayer Configuration: Design of a New Class of Amperometric Biosensors. Anal. Chem..

[20] Campuzano S., Glávez R., Pedrero M., de Villena F.J.M., Pingarrón J.M. (2002). Preparation, characterization and application of alkanethiol self-assembled monolayers modified with tetrathiafulvalene and glucose oxidase at a gold disk electrode. J. Electroanal. Chem..

[21] Moscovici M., Bhimji A., Kelley S.O. (2013). Rapid and specific electrochemical detection of prostate cancer cells using an aperture sensor array. Lab Chip.

[22] Jiang L., Glidle A., Griffith A., McNeil C.J., Cooper J.M. (1997). Characterising the formation of a bioelectrochemical interface at a self-assembled monolayer using X-ray photoelectron spectroscopy. Bioelectrochem. Bioenerg..

[23] Chen Y., Guo L.-R., Chen W., Yang X.-J., Jin B., Zheng L.-M., Xia X.-H. (2009). 3-Mercaptopropylphosphonic Acid Modified Gold Electrode for Electrochemical Detection of Dopamine. Bioelectrochemistry.

[24] Bourg M.C., Badia A., Lennox R.B. (2000). Gold-sulfur bonding in 2D and 3D self-assembled monolayers: XPS characterization. J. Phys. Chem. B.

[25] Mikhlin Y., Likhatski M., Tomashevich Y., Romanchenko A., Erenburg S., Trubina S. (2010). XAS and XPS examination of the Au-S nanostructures produced via the reduction of aqueous gold(III) by sulfide ions. J. Electron Spectrosc. Relat. Phenom..

[26] Wu J., Campuzano S., Halford C., Haake D.A., Wang J. (2010). Ternary surface monolayers for ultrasensitive (Zeptomole) amperometric detection of nucleic acid hybridization without signal amplification. Anal. Chem..

[27] Janek R.P., Fawcett W.R., Ulman A. (1998). Impedance Spectroscopy of Self-Assembled Monolayers on Au(111): Sodium Ferrocyanide Charge Transfer at Modified Electrodes. Langmuir.

[28] Song S.Y., Yoon H.C. (2009). Boronic acid-modified thin film interface for specific binding of glycated hemoglobin (HbA1c) and electrochemical biosensing. Sens. Actuators B Chem..

